# Complications after CD19+ CAR T-Cell Therapy

**DOI:** 10.3390/cancers12113445

**Published:** 2020-11-19

**Authors:** Olaf Penack, Christian Koenecke

**Affiliations:** 1 Department for Hematology, Oncology and Tumorimmunology, Charité Universitätsmedizin Berlin, Campus Virchow Clinic, Augustenburger Platz, 113353 Berlin, Germany; 2 Department of Hematology, Hemostasis, Oncology and Stem Cell Transplantation, Hannover Medical School, Carl-Neuberg-Str. 1, D-30625 Hannover, Germany; koenecke.christian@mh-hannover.de

**Keywords:** chimeric antigen receptor, CAR T-cells, toxicity, complications, cytokine release syndrome, CD19, lymphoma, leukemia

## Abstract

**Simple Summary:**

CD19+ Chimeric antigen receptor (CAR) T-cells are used against CD19+ hematologic malignancies, such as high-grade B-cell lymphoma and acute lymphoblastic leukemia. Since this is a relatively new treatment approach, not all potential side effects are well described, and the underlying pathobiology is often not well defined. Here, we summarize current data on the incidence and the current management of CD19+ CAR T-cell complications. We discuss frequently occurring toxicities and we highlight evidence for the occurrence of rarer side effects affecting different organ systems. In addition, we highlight new findings that shed light on the pathophysiology of CAR T-cell-related complications.

**Abstract:**

Clinical trials demonstrated that CD19+ chimeric antigen receptor (CAR) T-cells can be highly effective against a number of malignancies. However, the complete risk profile of CAR T-cells could not be defined in the initial trials. Currently, there is emerging evidence derived from post approval studies in CD19+ CAR T-cells demonstrating both short-term and medium-term effects, which were unknown at the time of regulatory approval. Here, we review the incidence and the current management of CD19+ CAR T-cell complications. We highlight frequently occurring events, such as cytokine release syndrome, immune effector cell-associated neurotoxicity syndrome, cardiotoxicity, pulmonary toxicity, metabolic complications, secondary macrophage-activation syndrome, and prolonged cytopenia. Furthermore, we present evidence supporting the hypothesis that CAR T-cell-mediated toxicities can involve any other organ system and we discuss the potential risk of long-term complications. Finally, we discuss recent pre-clinical and clinical data shedding new light on the pathophysiology of CAR T-cell-related complications.

## 1. Background

CAR-T cells that target CD19 have become standard treatments of relapsed or refractory hematological malignancies, such as B cell acute lymphoblastic leukemia (ALL) and relapsed or refractory large B cell lymphoma (LBCL). In addition, numerous trials in further tumor entities are under way, making it likely that in the near future, there will be a broader clinical use of CAR-T cells with different antigen specificities.

The safety of CAR-T cells is of major concern, since this new class of antitumor therapy has previously unknown side effects. The complete risk profile of CD19+ CAR T-cells could not be defined in the relatively small initial trials leading to approval of CAR T-cell products. This led to the obligation to document toxicities in any patient receiving commercial CAR T-cell products.

Currently, there is emerging evidence demonstrating both short-term and medium-term effects, which were in part unknown at the time of regulatory approval. However, a major hurdle towards a better understanding is our incomplete knowledge on the underlying pathophysiology of some of those complications. A challenge in the clinic is to predict the timelines of unwanted effects because of the ability of CAR-T cells to persist and expand in vivo. Another challenge will be to determine if the to-date detected side effects of CD19-targeting CAR T-cells may also apply to future CAR T-cell therapies (CART) targeting other antigens. In addition, the toxicity profile of future third- or fourth-generation CAR T-cell products has not been well defined. Second-generation CAR constructs use one costimulatory signaling domain, whereas in third-generation CAR constructs, two costimulatory domains are combined. In fourth-generation CAR constructs, further genes are expressed, which may facilitate the activation of CAR T-cells. Therefore, there is the possibility that third- or fourth-generation CAR T-cell products have additional toxicities or more severe forms of similar toxicities. However, early data suggest that the toxicities may be lower as compared with the current approved second-generation CAR-T products [1]. In this article, we discuss the available data on toxicities of the commercially available second-generation CD19-targeting CAR T-cell products and give an outlook on the expected toxicities of emerging CART (e.g., for multiple myeloma and solid tumors).

## 2. Cytokine Release Syndrome 

In response to contact with the target antigen, signaling through the CAR induces CAR T-cell proliferation and cytokine production. This leads to a cascade of immune stimulation involving the activation of macrophages. In a xenogeneic model of CRS using immunocompromised mice injected with human CD19+ lymphoma cells as well as CD19+ CAR-T cells, a critical role of macrophage-derived cytokines, such as IL-6, IL-1, and nitric oxide, has been demonstrated [2]. However, multiple inflammatory proteins are elevated in peripheral blood during CRS, including C-reactive protein, ferritin, interferon-γ, interleukins (IL-1, IL-2Rα, IL-6, IL-8, IL-10, IL-15), monocyte inflammatory proteins, monocyte chemoattractant proteins, and tumor necrosis factor receptors.

An important step of the inflammatory cascade seems to be the activation of endothelial cells in the microvasculature of different organs, resulting in capillary leakage. In patients with severe CRS, angiopoietin-2 and serum von Willebrand factor were significantly increased. Moreover, both factors were predictive of severe CRS when assessed prior to CAR-T cell therapy [3]. Other risk factors for the development of CRS include high CAR T-cell and tumor cell numbers as well as low platelet counts, comorbidities, and early onset of CRS [3,4,5,6]. Moreover, the costimulatory capabilities as well as the antigen binding characteristics of the CAR construct are likely to determine the CRS risk [7]. The reported incidences of CRS are quite variable, with ranges between 30% and 100%, not only depending on the presence of risk factors in the patient cohorts but also on different CRS grading systems used in the literature [8,9,10]. The incidences of CRS reported in the literature are summarized in Table 1. In accordance, the incidence of severe CRS (grades 3–4) has been reported to be in the range from 10–30% [8,9]. Of note, more recent publications report lower incidences of severe CRS and lower rates of intensive care unit treatments, especially in more recent CAR T cell products, which are therefore applied in an outpatient setting [11]. Interestingly, in a recently reported phase 1/2 study, a bicistronic CAR targeting CD19 and CD22 followed by an anti-PD1 showed no induction of ≥°III CRS [12]. 

In established CAR T cell products, this fact probably derives from improved management of CRS, namely from an earlier start of effective (steroid) treatment, which was also shown in cohort 4 of the ZUMA-1 study [13].

Recently, CD19-CAR-T cell products have been being tested in indolent B-NHL, such as marginal zone lymphoma and follicular lymphoma, with remarkably high anti-lymphoma activity. However, the frequency and severity of CRS was similar to that in LBCL [14].

The typical onset of CRS is between day + 3 and +6 after CAR T-cell infusion, with clinical manifestations ranging from mild pyrexia through severe cases of multi-organ dysfunction. Grading of CRS is recommended using the ASTCT grading system, which is also supported by the EBMT (Table 2) [9,15]. Pharmacologic management of CRS is adapted to the respective CRS grades [9,15].

**Grade 1:** Symptomatic treatment (e.g., anti-pyretics and fluids), considering broad-spectrum antibiotics. In case of very high fiver or persistence for more than three days, consider Tocilizumab.

**Grade 2:** In addition, Tocilizumab 8 mg/kg (max. 800 mg), which can be repeated every 8 h for a maximum of four administrations.

**Grade 3:** In addition, Dexamethasone 10–20 mg, which can be repeated every 6 h. 

**Grade 4:** Methylprednisolone 1000 mg/day in exchange for Dexamethasone.

In refractory cases, other immunosuppressive agents, such as Siltuximab (anti IL-6) and Anakinra (anti-IL-1 receptor) [16], as well as cytokine absorption methods, have been used successfully [17]. Grades 3 to 4 CRS are managed in intensive care units and standard approaches for cardiovascular and pulmonary support are used.

Of note, a number of CART complications, which are depicted below, are associated or even induced by higher grades of CRS. Table 3 summarizes prevention and therapy strategies for different CD19+ CAR T-cell related complications. 

## 3. Immune Effector Cell-Associated Neurotoxicity Syndrome (ICANS) 

The pathophysiology of ICANS is not well understood mainly because reliable animal models are lacking and because patient material for research is often not available. However, in peripheral blood, a multitude of inflammatory mediators are elevated, similar to the situation in CRS [21,22]. In ICANS, the vascular pathobiology seems to play a central role, as endothelial activation and multifocal vascular disorder have been found in the brain of patients with fatal neurotoxicity. In addition, biomarkers for endothelial activation were predictive of neurotoxicity even before CAR T-cell administration [23]. Another potentially relevant recent finding is that mural cells, which surround the cerebral endothelium and are critical for blood–brain barrier integrity, express CD19. This may help in explaining the increased frequency of ICANS in patients receiving CD19-targeting immunotherapies [24]. It remains to be determined if future CAR T-cell therapies targeting other antigens will have a lower incidence of ICANS. Currently, the most important risk factor for the development of ICANS is the presence of severe CRS. Other risk factors include a high tumor burden and preexisting neurologic disorders [8,21,22].

The reported incidences are variable in a range between 10% and 50% (summarized in Table 1). Recent data showed a similar frequency of ICAN for other B-cell malignancies, such as indolent B-NHL or mantle-cell lymphoma [14,25].

The time to onset of ICANS-related symptoms is more variable as compared to the onset of CRS and was reported to be in a range of 1–34 days after CAR T-cell infusion [6,18,26,27]. Clinical presentation often starts with a deterioration in handwriting and later on, non-specific symptoms, such as disturbances of attention, agitation, confusion, tremors, headaches, or seizures, may occur. The mental status can be disturbed, ranging from minor changes to severe disturbances (e.g., coma). Regularly, ICANS is reversible; however, infrequently, fatal cases due to hemorrhage or cerebral edema may occur [28]. Grading of ICANS is performed according to the recommendations of ACTCT and EBMT as shown in Table 4 [9,15]. The backbone of pharmacologic management of ICANS is steroids according to the respective ICANS grades [9,15]. Grade 1 ICANS is typically not treated with steroids. Dexamethasone (e.g., 10 mg every 6 h) is generally used in ICANS grade 2–3 whereas high-dose methylprednisolone (e.g., 1000 mg) is often used in grade four ICANS. Steroids are tapered over days to several weeks but there is a risk of recurrence of ICANS [29]. In case of refractory ICANS despite high-dose steroids, there is no standard clinical approach.

## 4. Complications after CAR T-Cell Therapy Other than CRS or ICANS

Initial publications derived from the pivotal trials reported a limited number of patients (<100 in most trials) with a limited observation period. However, in the last two to three years, many more patients were treated with CAR T-cells, both with approved drugs and in clinical trials. In the US-based CIBMTR database already >2000 patients and in the EBMT database nearly 1000 patients with CAR T-cell therapy were included (current figures given at cibmtr.org and ebmt.org). This explains why additional complications occurring at later time points or occurring less frequently were reported, including neurologic events, cardiotoxicity, pulmonary toxicity, metabolic complications, secondary macrophage-activation syndrome, prolonged cytopenia, certain infections, immune-related events, and subsequent malignancies. Table 5 summarizes the published literature on the incidence of CAR-T-related complications other than CRS and ICANS.

## 5. Late Neurologic and Psychiatric Events

In a recent published report, about 10% of patients surviving CAR T-cell therapy longer than three months had neurological events other than ICANS, including ischemic attacks, peripheral neuropathy, and Alzheimer’s dementia [30]. The patho-mechanism behind this association is not known. Furthermore, psychiatric events have been detected in 9% of patients undergoing CAR T-cells in that study. However, 50% of those patients had a pre-existing psychiatric disorder [30]. It is not clear whether such side effects are directly or indirectly associated with CAR T-cells, since pathophysiologic mechanisms for these side effects are unclear and no sufficient control patients were included in those analyses.

## 6. Cardiovascular Toxicities

Cardiovascular complications have been initially reported in children treated with CAR T-cells for ALL. In the ELIANA trial, grade 3 toxicities of cardiovascular origin were hypotension, fluid overload, and pulmonary edema in more than 5% of patients [6]. Additionally, cardiomyopathy with left ventricular systolic dysfunction was detected in additional retrospective analyses. However, such complications were reversible in most children weeks to months after CAR T-cells [33,34,35].

In the adult patient population, at least two retrospective analyses were published for approved CAR T-cell products. In one study, major cardiovascular events occurred in 17% of patients till one month after CAR T-cell infusion [36]. In another retrospective monocentric study of 60 consecutive adult patients with LBCL, who were treated either with axicabtagene ciloleucel or tisagenlecleucel, 48 cardiovascular adverse events were observed in 32 patients within one year after infusion [32]. Similar to the cardiovascular toxicities seen in the pediatric population, hypotension and fluid retention were most common. Atrial fibrillation and hypertension were additional cardiovascular side effects in adults. Of note, most cardiovascular events were detected in patients also developing CRS [32,36]. The prevailing patho-mechanism seems to be the exacerbation of pre-existing cardiovascular damage caused by CRS-related stress. 

## 7. Pulmonary Toxicity

Pulmonary toxicities are complications of special interest in the field of immunotherapies, especially for checkpoint inhibitor therapies. In CAR T-cell therapy recipients, pulmonary toxicities were manageable in most of the cases and no unsuspected lung toxicity occurred to date. However, pulmonary complications are also more frequent in patients with higher grade CRS [32]. The most frequent pulmonary symptom was hypoxia, but also pleural effusion, pulmonary embolism, allergic rhinitis, and pneumomediastinum were described [32]. To date, there is no comprehensive analysis for lung toxicity in recipients of CAR T-cell therapy, especially long-term follow-up with consecutive lung function tests, including transfer capacity of the lung, which are currently missing.

## 8. Metabolic Complications

In a recent study with 60 patients by Wudikarn and colleagues, metabolic toxicities after CART were detected as a frequent complication [32]. Typical electrolyte abnormalities were hypokalemia, hypophosphatemia, and hypocalcemia. Hyperglycemia and hypoglycemia were also frequent [19,20,32]. However, such complications are manageable, and persistence is not expected.

## 9. Secondary Macrophage-Activation Syndrome (sHLH/MAS)

A recent poll by the EBMT reported an estimated incidence of sHLH/MAS between 3% and 4% [37]. However, it is difficult to dissect secondary macrophage-activation syndrome after CART from severe CRS because some of the diagnostic criteria are overlapping, leading to low practicability of the conventional diagnostic criteria [37]. A reasonable solution that has been proposed recently is that the diagnosis of sHLH/MAS should be made in case of very high ferritin levels (e.g., >10,000 ng/mL) in addition to the typical symptoms [38]. The management of sHLH/MAS after CAR T-cell therapy is currently a matter of debate, but Tocilizumab and steroids are considered the therapeutic backbone. Whether additional agents have positive effects is not yet clear. One opportunity is to administer etoposide in reference to HLH/MAS management outside the setting of CAR T-cell therapy [39]. Another option is to use Emapalumab, an anti-interferon-gamma antibody, which is highly upregulated during MAS/HLH [40]. 

## 10. B-Cell Aplasia

Single infusion with CD19-directed CAR T-cell products induces B-cell aplasia associated with hypogammaglobulinemia, which is shown in numerous studies [6,41]. In most patients, immunoglobulin replacement was used to prevent infectious complications. However, currently, it is not clear whether replacement therapy adds to outcome/improved quality of life in CD19 CAR T-cell patients [42]. Persistence of CD19-directed CAR T-cells will most probably maintain B-cell aplasia. However, long-term studies will clarify whether CD19 CAR T-cell products will be active in the recipient’s body for years. Whether B-cell aplasia or hypogammaglobulinemia will be of concern in CART of other B-lineage target epitopes, such as BCMA, will be of interest.

## 11. Prolonged Cytopenia

Early, late, and prolonged hematotoxicity after CAR T-cells occur in different CAR constructs and are the most common adverse events after lymphodepletion and CAR T-cell transfusion, with neutropenia being the most frequent. Early cytopenia is frequent in >80% of patients, whereas late cytopenia occurs in 30–40%. In a recent publication on late effects after CAR T-cell therapy, a persisting (after day + 90) cytopenia rate of 16% was described [30]. Overall, between 8% and 18% of CAR T-cell recipients were reported to have prolonged cytopenia. Lymphopenia is seen in 35% of patients at 1 year after anti-CD19 CAR T-cell therapy. One study described that late hematologic toxicity is most frequent in patients with CRS and in patients with a history of stem cell transplantation [43]. In patients with relapsed or refractory large B-cell lymphoma treated with axicabtagene ciloleucel (ZUMA-1 and ZUMA-9 trials), 48% of patients had grade 3–4 cytopenia at day 30 (anemia 16%, neutropenia 29%, and thrombocytopenia 42%). After two years, grade 3–4 cytopenia persisted in one out of nine evaluable patients (11%) [31]. It is largely unknown which factors contribute to cytopenia, e.g., insufficient or late hematopoietic recovery after CART. In one study, 83 patients receiving CD19 CART were evaluated for hematopoietic recovery. In this cohort, baseline cytopenia, higher grades of CRS or ICANS, and higher peak of C-reactive protein or ferritin were associated with a lower likelihood of complete count recovery [44]. Disease entities play a minor role, since cytopenia is observed in different entities, such as myeloma, ALL, and lymphoma. The use of G-CSF might be beneficial in patients without signs of cytokine release syndrome and prolonged cytopenia.

## 12. Infections and Vaccinations

It has been reported that in patients surviving day+90 after CAR T-cell therapy, late infections occurred in approximately 60% of patients. Of those infections, 60% were bacterial infections, 31% were viral infections (mostly respiratory viruses), and 9% were fungal infections [30]. In another study, 60 patients with LBCL were retrospectively analyzed for incidence, risk factors, and management of infections [45]. In total, 101 infections were observed in this patient cohort, among them, 23 severe, one life-threatening, and one lethal. The cumulative incidence of infections at one year was 63.3%. Most infections were bacterial (57.2%), severe bacterial (29.6%), viral (44.7%), and fungal (4%). The use of corticosteroids for management of CRS or ICANS was associated with an increased risk of infections. It is recommended to use antimicrobial, antiviral, and antifungal prophylaxis in patients after CART, not only in cytopenic patients. Most centers use Cotrimoxazol Trimethoprim and Aciclovir until approximately 6 months after CD19+ CAR T-cell infusion. However, antifungal therapy, e.g., with Posaconazole, could also be considered.

Data on infection in the context of COVID-19 and CART are sparse. However, it was recently reported that in a cohort of 77 patients with SARS/COV-2 infection receiving cellular therapy (autologous, allogeneic, and CART), a favorable outcome was reported. Overall survival at one month after cellular therapy was 78% [46]. This data suggest that COVID-19 infections in CAR T cell recipients might be manageable. Nevertheless, robust data in a larger cohort is pending.

It is a matter of debate if, and when, patients after CD19 CAR T-cell therapy should be vaccinated as these patients may have low vaccine responses. On the other hand, these patients often have considerable risk for infections and the vaccination efficacy does not exclusively depend on CD19+ B-cell responses. In fact, patients after CD19+ CAR T-cell therapy may have persistence of plasma cells and may be able to have adequate vaccine responses [47]. Therefore, the administration of re-vaccination should be considered in all recipients of CAR T-cell therapy. We suggest performing seasonal influenza A vaccinations, also for close relatives of the patient. Beginning at 6 months after CAR T-cell infusion, a comprehensive re-vaccination boost can be started; however, live attenuated vaccines should not be administered within the first year [48]. Recommended vaccinations are similar to the re-vaccination schedule after allogeneic stem cell transplantation [49]. Of note, the assessment of anti-infectious antibody levels (titers) in patients after substitution of immunoglobulins is misleading and not recommended.

## 13. Immune-Related Events and GVHD

In a study on later effects after CAR T-cell therapy, an incidence of 8% of immune-related events was reported, including alveolitis, pneumonitis, persistent skin rash, collagenous colitis, and persistent flu-like syndrome [30]. Generally, it is not expected that autologous or allogeneic donor-derived CAR T cells induce or trigger GVHD or GVHD-like symptoms [50]. However, in another study, out of 15 patients receiving CAR T-cells after previous allogeneic HCT, 3 patients (20%) developed GVHD requiring systemic therapy [30]. This is an important side effect, which might be even more frequent when third-party CAR T cell products are introduced, unless the endogenous T cell receptor is genetically deleted or inactivated. Taken together, GVHD might be a side effect of special interest and if suspected standard therapy is recommended [9].

## 14. Subsequent Malignancies

In a study on late effects after CD19 CAR T-cell therapy with a median duration of follow-up of around 28 months, 21 out of 86 patients had ongoing CR. Six of those patients (29%) developed subsequent malignant disease (2 MDS, 1 melanoma, 2 non-melanoma skin cancer, and 1 multiple myeloma) [30]. However, a follow-up period of 2.5 years is most probably not sufficient to estimate the frequency of secondary malignancies after CART, since the development of malignancies is typically associated with latency [51]. Furthermore, it will be difficult to evaluate this risk independently of cytostatic therapy before CART.

## 15. Potential Risk of other Long-Term Complications and Implications of the Available Evidence for Clinical Care 

Since CAR T-cell therapy is based on genetic manipulation of recipients’ lymphocytes, one has to be aware of genotoxicity, such as lymphoma induction/tumor induction, itself [52], To date, this long-term risk cannot be estimated and it is important to perform long-term surveillance. In the future routine checkups for monitoring and prevention, unexpected or rare organ toxicities will be necessary. Data on CAR T cell persistence will also be of major importance to estimate the risk of long-term organ toxicities. Such recommendations will be based on mature outcome data, years after the approval of CART.

## 16. Outlook and Future Perspectives

A major future effort will be to reduce CAR T-cell treatment-related toxicities. There are numerous disease- and patient-related variables that contribute to the risk of toxicities, such as co-morbidities, age, tumor type, tumor cell burden, and conditioning therapy. However, modifications in the CAR T-cell product characteristics offer the most promising perspective to decrease toxicities. Specifically, the design of optimized CAR constructs may lead to the opportunity to reduce toxicities. This review is limited by the fact that we mainly focused on two products (Tisa-cel and Axi-cel) in which long-term data/post approval data was available. A variety of new CART are underway, for LBL, such as Liso-cel, mantle cell lymphoma Brexucabtagen autoleucel, and Ide-cel, orva-cel, and the JNJ-4528 for multiple myeloma. Furthermore, a recent report describes the generation of a CD19 41BB construct with a lower production of cytokines and slower proliferation kinetic. No significant elevation in serum cytokine levels after CAR T cell infusion were detected and no CRS or neurotoxicity occurred in the 11 patients treated with this construct [53]. Recently, data on allogeneic CD19 CAR-T cell constructs with an eliminated endogenous T cell receptor were reported. Safety data on this third party off the shelf CART was encouraging, since no GVHD occurred and ≥°III CRS/ICAN rates were rather low [54].

In addition, a better understanding of the pathophysiology of CAR T-cell-related complications will be critical. Animal models that were developed recently could be the key to a better understanding [2,55]. The development of therapies aimed at the normalization of endothelial dysfunction, which is associated to CAR T-cell-related complications, may be specifically promising [3,23,56].

A major concern regarding CART outside the CD19 setting is on-target off-tumor toxicities. The difficulty is that there is a lack of suitable tumor neo-antigens and the majority antigens that are under investigation in CAR T-cells trials have a considerable expression in normal tissues. As a result, the use of CAR-T cells for the treatment of solid tumors may have an increased risk of severe toxicities as compared with CD19-specific CAR T-cells [57,58].

## 17. Conclusions

CD19+ CAR T-cells are promising new treatment options for patients with certain types of leukemia and lymphoma. However, there are considerable toxicities, such as cytokine release syndrome, immune effector cell-associated neurotoxicity syndrome, cardiotoxicity, pulmonary toxicity, metabolic complications, secondary macrophage-activation syndrome, and prolonged cytopenia. Optimization of the management of these unwanted short-term and medium-term effects could further improve clinical outcome. 

## Figures and Tables

**Table 1:** Incidence of non-relapse mortality (NRM), cytokine-release syndrome (CRS), and Immune Effector Cell-Associated Neurotoxicity Syndrome (ICANS) in patients included in key clinical trials with commercially approved CAR T-cells (Tisa-cel and Ax-cel).

Reference Year	Product	Patients (*n*)	NRM	CRS	ICAN
Phase I/IIa 2014 [6]	Tisa-Cel	30	0%	All patients (100%) developed CRS 27% developed severe CRS requiring hemodynamic support	43% of patients developed ICANS ranging from delirium to global encephalopathy
Phase II ELIANA 2018 [18]	Tisa-Cel	75	8%	77% of patients developed CRS of any grade 47% required ICU admission 25% required high-dose vasopressorsupport	40% of patients developed ICANS of any grade 13% grade 3 ICANS
Zuma-1 [19]	Axi-Cel	101	4%	Grade 3 or worse CRS occurred in 11% of patients	Grade 3 or worse neurological events in 32% of patients
Juliet [20]	Tisa-Cel	93	0%	Grade 3 or worse CRS occurred in 22% of patients	Grade 3 or worse neurological events in 12% of patients
Real world (PMID: 32667831)	Axi-Cel	122	6%	Grade 3 or worse occurred in 16% of patients	Grade 3 or worse neurological events in 35%

**Table 2:** Suggested CRS grading, modified ASTCT consensus statement supported by the EBMT recommendations [9,15].

CRS Parameter	Grade 1	Grade 2	Grade 3	Grade 4
Temperature ≥ 38 °C	Yes	Yes/#	Yes/#	Yes/#
with				
Hypotension	No	Not requiring a vasopressor	Requiring one vasopressor	Requiring more than one vasopressor
and/or +				
Hypoxia	No	Requiring low-flow oxygen	Requiring high-flow oxygen	Requiring positive pressure (CPAP, BiPAP, mechanical ventilation)

**#** In patients receiving antipyretic or anti-inflammatory therapy (e.g. tocilizumab or steroids) fever is not required to grade CRS severity. + total CRS grade is determined by the highest CRS parameter grading.

**Table 3:** Recommendations on prevention as well as comments on the management of toxicities in patients receiving anti-CD19 CAR T-cells.

Complication	Prevention	Management/Comments
CRS	Reduce tumor cell numbers prior to infusion Limit the number of infused CAR T-cells	Antipyretics, Fluids, Toziclizumab, Steroids
ICANS	Reduce tumor cell numbers prior to infusion Limit the number of infused CAR T-cells Neurologic assessment and treatment of neurologic diseases prior to CAR T-cell infusion	Steroids
Pulmonary complications	Lung function tests before CAR T-cell infusion to assess the risk	Non-invasive ventilation
Cardiovascular toxicity	Avoid cardiotoxic chemotherapy before CAR T-cell Echo to assess cardiac function	Reduce cardiovascular risk factors in long term survivors
Secondary macrophage-activation syndrome	Unknown how it can be prevented	Hard to differentiate from severe CRS Etoposide, Emapalumab possible drugs
B-cell aplasia	Cannot be prevented	IgG Substitution
Prolonged cytopenia	Preventive measures unknown	Role of growth factor substitution unclear
Infections	Protected environment during neutropenic phase	Anti-bacterial prophylaxis during neutropenic phase PJP and herpes prophylaxis till approximately 6 months post CAR T Antifungal prophylaxis not standard

**Table 4:** Suggested ICANS grading, modified ASTCT consensus statement supported by the EBMT recommendations [9,15]. The total ICANS grade is determined by the most severe neurotoxicity domain grading.

Neurotoxicity Domain.	Grade 1	Grade 2	Grade 3	Grade 4
ICE Score ^+^	9–7	6–3	2–0	0 unable to perform ICE
Depressed level of consciousness	Awakens spontaneously	Awakens to voice	Awakens only to tactile stimulus	Patient is unarousable or requires vigorous or repetitive tactile stimuli to arouse. Stupor or coma.
Seizure	None	None	Seizure not fulfilling criteria for grade 4	Life-threatening prolonged seizure (>5 min); or Repetitive clinical or electrical seizures without return to baseline in between
Motor findings	None	None	None	Deep focal motor weakness such as hemiparesis or paraparesis
Elevated ICP/cerebral edema	None	None	Focal/local edema on Neuroimaging ^#^	Diffuse cerebral edema on neuroimaging; decerebrate or decorticate posturing; or cranial nerve VI palsy; or papilledema; or Cushing’s triad

^+^ ICE Score (max 10): (1) Orientation (4 points): time (year, month), place (city, hospital); (2) Naming (3 points): ability to name three standard objects of daily life; (3) Writing (1 point): ability to write standard sentence; (4) Attention (1 point): ability to count backward; (5) Following commands (1 point): ability to follow simple commands. **^#^** Intracranial hemorrhage with or without associated edema is not considered a neurotoxicity feature and is excluded from ICANS grading.

**Table 5:** CAR-T-related complications (other than CRS and ICANS) reported in the literature. Blank spaces where left for parameters not reported in the respective publications. NHL = Non-Hodgins Lymphoma, CLL–Chronic Lymphocytic Leukemia.

Reference Year	Product Disease	Patients (*n*) Trial vs. Real World	Neurologic	Pulmonary	Cardiologic	Metabolic	Prolonged Cytopenia	Infection	Immune-related	Secondary Tumor	Other
Phase II ELIANA 2018 [18]	Tisa-Cel	75 trial		Hypoxia 24%		Hypokalemia 27% Hypophos-phatemia 24%	37% at day 28	Febrile neutro-penia 35% till week 8			Tumor lysis syndrome 4% Aspartate-aminotransferase increased 27% Bilirubin increased 17%
Cordeiro et al. [30]	ALL NHL CLL	86 trial	10%				16% after day + 90	61%	8%	29% of patients in continuous CR	*Hypogammaglobulinemia* (IgG < 400 mg/dL or i.v immunoglobulinm (IVIG) replacement, observed in 67%
Zuma-1 and -9 [31]	LBCL	31 trial	Fatigue 53% Headache 46% Confused state 27% Dizziness 21% Somnolece 17%	Hypoxia 31% Cough 29% Dyspnea 21% Pleural effsuion 16%	Hypotension 58% Tachycardia 40% Peripheral edema 19% Tachycardia 19% Hyper-tension 16%	Hypocalcemia 40% Hyponataemia 35% Hypokalemia 33% Hypophos-phatemia 29% Hyperglycemia 19% Hypomagnes-emia 19%	48% by day + 30 11% at 2 years	28% grade II or worse			
Juliet [20]	LBCL	93 trial	Dizziness 13% Anxiety 12% Fatigue 28%	Dyspnea 19% Cough 19%	Hypotension 29% Tachycardia 12% Peripheral edema 17%	Hypokalemia 23% Hypomagnes-emia 19%, Hypophosphatemia 19%	D + 28 32%	D + 28 20% infections			
MSKCC [32]	NHL ALL	60 Real world	18% at 1y	15% at 1y	17% at 1y	55% at 1y	58% at 1y	35% at 1y			Hepatic 25% at 1y

## References

[1] Cao G., Lei L., Zhu X. (2019). Efficiency and safety of autologous chimeric antigen receptor T-cells therapy used for patients with lymphoma: A systematic review and meta-analysis. Medicine.

[2] Giavridis T., Van der Stegen S.J.C., Eyquem J., Hamieh M., Piersigilli A., Sadelain M. (2018). CAR T cell-induced cytokine release syndrome is mediated by macrophages and abated by IL-1 blockade. Nat. Med..

[3] Hay K.A., Hanafi L.A., Li D., Gust J., Liles W.C., Wurfel M.M., Lopez J.A., Chen J., Chung D., Harju-Baker S. (2017). Kinetics and biomarkers of severe cytokine release syndrome after CD19 chimeric antigen receptor-modified T-cell therapy. Blood.

[4] Teachey D.T., Lacey S.F., Shaw P.A., Melenhorst J.J., Maude S.L., Frey N., Pequignot E., Gonzalez V.E., Chen F., Finklestein J. (2016). Identification of predictive biomarkers for cytokine release syndrome after chimeric antigen receptor t-cell therapy for acute lymphoblastic leukemia. Cancer Discov..

[5] Davila M.L., Riviere I., Wang X., Bartido S., Park J., Curran K., Chung S.S., Stefanski J., Borquez-Ojeda O., Olszewska M. (2014). Efficacy and toxicity management of 19-28z CAR T cell therapy in B cell acute lymphoblastic leukemia. Sci. Transl. Med..

[6] Maude S.L., Frey N., Shaw P.A., Aplenc R., Barrett D.M., Bunin N.J., Chew A., Gonzalez V.E., Zheng Z., Lacey S.F. (2014). Chimeric antigen receptor T cells for sustained remissions in leukemia. N. Engl. J. Med..

[7] Ghorashian S., Kramer A.M., Onuoha S., Wright G., Bartram J., Richardson R., Albon S.J., Casanovas-Company J., Castro F., Popova B. (2019). Enhanced CAR T cell expansion and prolonged persistence in pediatric patients with ALL treated with a low-affinity CD19 CAR. Nat. Med..

[8] Pennisi M., Jain T., Santomasso B.D., Mead E., Wudhikarn K., Silverberg M.L., Batlevi Y., Shouval R., Devlin S.M., Batlevi C. (2020). Comparing CAR T-cell toxicity grading systems: Application of the ASTCT grading system and implications for management. Blood Adv..

[9] Yakoub-Agha I., Chabannon C., Bader P., Basak G.W., Bonig H., Ciceri F., Corbacioglu S., Duarte R.F., Einsele H., Hudecek M. (2020). Management of adults and children undergoing chimeric antigen receptor T-cell therapy: Best practice recommendations of the European Society for Blood and Marrow Transplantation (EBMT) and the Joint Accreditation Committee of ISCT and EBMT (JACIE). Haematologica.

[10] Jacobson C.A., Hunter B.D., Redd R., Rodig S.J., Chen P.H., Wright K., Lipschitz M., Ritz J., Kamihara Y., Armand P. (2020). Axicabtagene ciloleucel in the non-trial setting: Outcomes and correlates of response, resistance, and toxicity. J. Clin. Oncol..

[11] Palomba M., Garcia J., Wang L., Dehner C., Chung K., Maloney D. (2018). TRANSCEND: Lisocabtagene maraleucel (liso-cel; JCAR017) healthcare resource utilization in patients with relapsed/refractory diffuse large B-cell lymphoma (DLBCL). Blood.

[12] Osborne W., Marzolini M., Tholouli E., Ramakrishnan A., Bachier C.R., McSweeney P.A., Irvine D., Zhang D., Al-Hajj M.A., Pule M.A. (2020). Phase I Alexander study of AUTO3, the first CD19/22 dual targeting CAR T cell therapy, with pembrolizumab in patients with relapsed/refractory (r/r) DLBCL. J. Clin. Oncol..

[13] Topp M., Van Meerten T., Houot R., Minnema M., Milpied N., Lugtenburg P., Thieblemont C., Wermke M., Song K., Avivi I. (2019). earlier steroid use with axicabtagene ciloleucel (Axi-Cel) in patients with relapsed/refractory large B cell lymphoma. Blood.

[14] Jacobson C.A., Chavez J.C., Sehgal A.R., William B.R., Munoz J., Salles G.A., Casulo C., Munshi P.N., Maloney D.G., De Vos S. (2020). Interim analysis of ZUMA-5: A phase II study of axicabtagene ciloleucel (axi-cel) in patients (pts) with relapsed/refractory indolent non-Hodgkin lymphoma (R/R iNHL). J. Clin. Oncol..

[15] Lee D.W., Santomasso B.D., Locke F.L., Ghobadi A., Turtle C.J., Brudno J.N., Maus M.V., Park J.H., Mead E., Pavletic S. (2019). ASTCT consensus grading for cytokine release syndrome and neurologic toxicity associated with immune effector cells. Biol. Blood Marrow Transpl..

[16] Brudno J.N., Kochenderfer J.N. (2019). Recent advances in CAR T-cell toxicity: Mechanisms, manifestations and management. Blood Rev..

[17] Stahl K., Schmidt B.M.W., Hoeper M.M., Skripuletz T., Mohn N., Beutel G., Eder M., Welte T., Ganser A., Falk C.S. (2020). Extracorporeal cytokine removal in severe CAR-T cell associated cytokine release syndrome. J. Crit. Care.

[18] Maude S.L., Laetsch T.W., Buechner J., Rives S., Boyer M., Bittencourt H., Bader P., Verneris M.R., Stefanski H.E., Myers G.D. (2018). Tisagenlecleucel in Children and Young Adults with B-Cell Lymphoblastic Leukemia. N. Engl. J. Med..

[19] Locke F.L., Ghobadi A., Jacobson C.A., Miklos D.B., Lekakis L.J., Oluwole O.O., Lin Y., Braunschweig I., Hill B.T., Timmerman J.M. (2019). Long-term safety and activity of axicabtagene ciloleucel in refractory large B-cell lymphoma (ZUMA-1): A single-arm, multicentre, phase 1-2 trial. Lancet Oncol..

[20] Schuster S.J., Bishop M.R., Tam C.S., Waller E.K., Borchmann P., McGuirk J.P., Jager U., Jaglowski S., Andreadis C., Westin J.R. (2019). Tisagenlecleucel in adult relapsed or refractory diffuse large B-cell lymphoma. N. Engl. J. Med..

[21] Chou C.K., Turtle C.J. (2020). Assessment and management of cytokine release syndrome and neurotoxicity following CD19 CAR-T cell therapy. Expert Opin. Biol..

[22] Santomasso B.D., Park J.H., Salloum D., Riviere I., Flynn J., Mead E., Halton E., Wang X., Senechal B., Purdon T. (2018). Clinical and biological correlates of neurotoxicity associated with CAR T-cell therapy in patients with B-cell acute lymphoblastic leukemia. Cancer Discov..

[23] Gust J., Hay K.A., Hanafi L.A., Li D., Myerson D., Gonzalez-Cuyar L.F., Yeung C., Liles W.C., Wurfel M., Lopez J.A. (2017). Endothelial activation and blood-brain barrier disruption in neurotoxicity after adoptive immunotherapy with CD19 CAR-T cells. Cancer Discov..

[24] Parker K.R., Migliorini D., Perkey E., Yost K.E., Bhaduri A., Bagga P., Haris M., Wilson N.E., Liu F., Gabunia K. (2020). Single-cell analyses identify brain mural cells expressing CD19 as potential off-tumor targets for CAR-T immunotherapies. Cell.

[25] Wang M., Munoz J., Goy A., Locke F.L., Jacobson C.A., Hill B.T., Timmerman J.M., Holmes H., Jaglowski S., Flinn I.W. (2020). KTE-X19 CAR T-cell therapy in relapsed or refractory mantle-cell lymphoma. N. Engl. J. Med..

[26] Gust J., Taraseviciute A., Turtle C.J. (2018). Neurotoxicity associated with CD19-targeted CAR-T cell therapies. CNS Drugs.

[27] Hunter B.D., Jacobson C.A. (2019). CAR T-cell associated neurotoxicity: Mechanisms, clinicopathologic correlates, and future directions. J. Natl. Cancer Inst..

[28] Landry K., Thomas A.A. (2020). Neurological complications of CAR T cell therapy. Curr. Oncol. Rep..

[29] Möhn N., Könecke C., Skripuletz T. (2020). Neurotoxizität unter CAR-T-Zell-Therapie (CAR: Chimärer Antigenrezeptor). DGNeurologie.

[30] Cordeiro A., Bezerra E.D., Hirayama A.V., Hill J.A., Wu Q.V., Voutsinas J., Sorror M.L., Turtle C.J., Maloney D.G., Bar M. (2020). Late events after treatment with CD19-targeted chimeric antigen receptor modified T cells. Biol. Blood Marrow Transpl..

[31] Strati P., Varma A., Adkins S., Nastoupil L.J., Westin J., Hagemeister F.B., Fowler N.H., Lee H.J., Fayad L.E., Samaniego F. (2020). Hematopoietic recovery and immune reconstitution after axicabtagene ciloleucel in patients with large B-cell lymphoma. Haematologica.

[32] Wudhikarn K., Pennisi M., Garcia-Recio M., Flynn J.R., Afuye A., Silverberg M.L., Maloy M.A., Devlin S.M., Batlevi C.L., Shah G.L. (2020). DLBCL patients treated with CD19 CAR T cells experience a high burden of organ toxicities but low nonrelapse mortality. Blood Adv..

[33] Alvi R.M., Frigault M.J., Fradley M.G., Jain M.D., Mahmood S.S., Awadalla M., Lee D.H., Zlotoff D.A., Zhang L., Drobni Z.D. (2019). Cardiovascular events among adults treated with chimeric antigen receptor T-cells (CAR-T). J. Am. Coll. Cardiol..

[34] Burstein D.S., Maude S., Grupp S., Griffis H., Rossano J., Lin K. (2018). Cardiac profile of chimeric antigen receptor T cell therapy in children: A single-institution experience. Biol. Blood Marrow Transpl..

[35] Fitzgerald J.C., Weiss S.L., Maude S.L., Barrett D.M., Lacey S.F., Melenhorst J.J., Shaw P., Berg R.A., June C.H., Porter D.L. (2017). Cytokine release syndrome after chimeric antigen receptor T cell therapy for acute lymphoblastic leukemia. Crit. Care Med..

[36] Lefebvre B., Kang Y., Smith A.M., Frey N.V., Carver J.R., Scherrer-Crosbie M. (2020). Cardiovascular effects of CAR T cell therapy: A retrospective study. JACC Cardiooncol..

[37] Sandler R.D., Tattersall R.S., Schoemans H., Greco R., Badoglio M., Labopin M., Alexander T., Kirgizov K., Rovira M., Saif M. (2020). Diagnosis and management of secondary HLH/MAS following HSCT and CAR-T cell therapy in adults. A review of the literature and a survey of practice within EBMT centres on behalf of the autoimmune diseases working party (ADWP) and transplant complications working party (TCWP). Front. Immunol..

[38] Neelapu S.S., Tummala S., Kebriaei P., Wierda W., Gutierrez C., Locke F.L., Komanduri K.V., Lin Y., Jain N., Daver N. (2018). Chimeric antigen receptor T-cell therapy—Assessment and management of toxicities. Nat. Rev. Clin. Oncol..

[39] Birndt S., Schenk T., Heinevetter B., Brunkhorst F.M., Maschmeyer G., Rothmann F., Weber T., Muller M., Panse J., Penack O. (2020). Hemophagocytic lymphohistiocytosis in adults: Collaborative analysis of 137 cases of a nationwide German registry. J. Cancer Res. Clin. Oncol..

[40] Henter J.I., Von Bahr Greenwood T., Bergsten E. (2020). Emapalumab in primary hemophagocytic lymphohistiocytosis. N. Engl. J. Med..

[41] Laetsch T.W., Myers G.D., Baruchel A., Dietz A.C., Pulsipher M.A., Bittencourt H., Buechner J., De Moerloose B., Davis K.L., Nemecek E. (2019). Patient-reported quality of life after tisagenlecleucel infusion in children and young adults with relapsed or refractory B-cell acute lymphoblastic leukaemia: A global, single-arm, phase 2 trial. Lancet Oncol..

[42] Misbah S.A., Weeratunga P. (2020). Immunoglobulin replacement and quality of life after CAR T-cell therapy. Lancet Oncol..

[43] Fried S., Avigdor A., Bielorai B., Meir A., Besser M.J., Schachter J., Shimoni A., Nagler A., Toren A., Jacoby E. (2019). Early and late hematologic toxicity following CD19 CAR-T cells. Bone Marrow Transpl..

[44] Jain T., Knezevic A., Pennisi M., Chen Y.X., Ruiz J.D., Purdon T.J., Devlin S.M., Smith M., Shah G.L., Halton E. (2020). Hematopoietic recovery in patients receiving chimeric antigen receptor T-cell therapy for hematologic malignancies. Blood Adv..

[45] Wudhikarn K., Palomba M.L., Pennisi M., Garcia-Recio M., Flynn J.R., Devlin S.M., Afuye A., Silverberg M.L., Maloy M.A., Shah G.N.L. (2020). Infection during the first year in patients treated with CD19 CAR T cells for diffuse large B cell lymphoma. Blood Cancer J..

[46] Shah G.L., DeWolf S., Lee Y.J., Tamari R., Dahi P.B., Lavery J.A., Ruiz J.D., Devlin S.M., Cho C., Peled J.U. (2020). Favorable outcomes of COVID-19 in recipients of hematopoietic cell transplantation. J. Clin. Investig..

[47] Bhoj V.G., Arhontoulis D., Wertheim G., Capobianchi J., Callahan C.A., Ellebrecht C.T., Obstfeld A.E., Lacey S.F., Melenhorst J.J., Nazimuddin F. (2016). Persistence of long-lived plasma cells and humoral immunity in individuals responding to CD19-directed CAR T-cell therapy. Blood.

[48] Hill J.A., Seo S.K. (2020). How I prevent infections in patients receiving CD19-targeted chimeric antigen receptor T cells for B-cell malignancies. Blood.

[49] Ullmann A.J., Schmidt-Hieber M., Bertz H., Heinz W.J., Kiehl M., Kruger W., Mousset S., Neuburger S., Neumann S., Penack O. (2016). Infectious diseases in allogeneic haematopoietic stem cell transplantation: Prevention and prophylaxis strategy guidelines 2016. Ann. Hematol..

[50] Anwer F., Shaukat A.A., Zahid U., Husnain M., McBride A., Persky D., Lim M., Hasan N., Bin Riaz I. (2017). Donor origin CAR T cells: Graft versus malignancy effect without GVHD, a systematic review. Immunotherapy.

[51] Ziegler A.K., Abend M., Port M., Dammann E., Homeyer R.S., Eder M., Ganser A., Schrem H., Koenecke C. (2019). Cumulative dosages of chemotherapy and radiotherapy exposure, and risk of secondary malignancies after allogeneic hematopoietic stem cell transplantation. Bone Marrow Transpl..

[52] Ruella M., Xu J., Barrett D.M., Fraietta J.A., Reich T.J., Ambrose D.E., Klichinsky M., Shestova O., Patel P.R., Kulikovskaya I. (2018). Induction of resistance to chimeric antigen receptor T cell therapy by transduction of a single leukemic B cell. Nat. Med..

[53] Ying Z., Huang X.F., Xiang X., Liu Y., Kang X., Song Y., Guo X., Liu H., Ding N., Zhang T. (2019). A safe and potent anti-CD19 CAR T cell therapy. Nat. Med..

[54] Neelapu S.S., Munoz J., Locke F.L., Miklos D.B., Brown R., McDevitt J.T., Mardiros A., Demirhan E., Konto C., Tees T.M. (2020). First-in-human data of ALLO-501 and ALLO-647 in relapsed/refractory large B-cell or follicular lymphoma (R/R LBCL/FL): ALPHA study. J. Clin. Oncol..

[55] Sterner R.M., Sakemura R., Cox M.J., Yang N., Khadka R.H., Forsman C.L., Hansen M.J., Jin F., Ayasoufi K., Hefazi M. (2019). GM-CSF inhibition reduces cytokine release syndrome and neuroinflammation but enhances CAR-T cell function in xenografts. Blood.

[56] Sun Y., Wang S., Zhao L., Zhang B., Chen H. (2019). IFN-gamma and TNF-alpha aggravate endothelial damage caused by CD123-targeted CAR T cell. Oncotargets Ther..

[57] Lamers C.H., Sleijfer S., Van Steenbergen S., Van Elzakker P., Van Krimpen B., Groot C., Vulto A., Den Bakker M., Oosterwijk E., Debets R. (2013). Treatment of metastatic renal cell carcinoma with CAIX CAR-engineered T cells: Clinical evaluation and management of on-target toxicity. Mol. Ther..

[58] Morgan R.A., Yang J.C., Kitano M., Dudley M.E., Laurencot C.M., Rosenberg S.A. (2010). Case report of a serious adverse event following the administration of T cells transduced with a chimeric antigen receptor recognizing ERBB2. Mol. Ther..

